# ABBV‐552 in patients with mild Alzheimer's disease: a randomized phase IIb trial

**DOI:** 10.1002/alz.70994

**Published:** 2025-12-26

**Authors:** Shau Yu Lynch, Jia Jia, Nick Miles, Joey Boiser, Derek L. Buhl, Ole Graff, Cindy Zadikoff

**Affiliations:** ^1^ Clinical Development AbbVie Inc. North Chicago Illinois USA; ^2^ Statistics AbbVie Inc North Chicago Illinois USA; ^3^ Clinical Pharmacology AbbVie Inc North Chicago Illinois USA; ^4^ Pharmacovigilance and Patient Safety AbbVie Inc. North Chicago Illinois USA; ^5^ Precision Medicine AbbVie Inc North Chicago Illinois USA

**Keywords:** ADAS‐Cog 14, Alzheimer's disease, Alzheimer's disease therapies, dementia, synaptic vesicle glycoprotein 2A

## Abstract

**INTRODUCTION:**

This proof‐of‐concept, dose‐finding phase IIb trial evaluated treatment with ABBV‐552 compared with placebo in participants with clinically diagnosed mild Alzheimer's disease (AD).

**METHODS:**

Participants aged 50 to 90 years with a Mini‐Mental State Examination score of 20 to 26 and a global Clinical Dementia Rating score of 0.5 to 1.0 were randomized 1:1:1:1 to placebo or ABBV‐552 (1, 5, or 15 mg) daily. The primary endpoint was the change from baseline in the 14‐item Alzheimer's Disease Assessment Scale‐Cognitive Subscale (ADAS‐Cog 14) at week 12.

**RESULTS:**

Two hundred sixty‐three participants were randomized. The least‐squares mean difference (vs placebo) in change from baseline at week 12 in ADAS‐Cog 14 total score (95% confidence interval) for ABBV‐552 1 mg was −0.02 (−1.87, 1.83), nominal *p *= 0.9819; 5 mg, −0.42 (−2.25, 1.42), nominal *p *= 0.6545; 15 mg, 0.25 (−1.58, 2.08), nominal *p *= 0.7860. Treatment‐emergent adverse events occurred in 48.5% of ABBV‐552 recipients versus 42.2% in the placebo group; no safety concerns were identified.

**DISCUSSION:**

ABBV‐552 did not demonstrate a meaningful difference versus placebo on the primary endpoint.

**Highlights:**

- ABBV‐552 is a small molecule that modulates the SV2A receptor in neurons
- ABBV‐552 may enhance synaptic efficiency leading to improved cognition in patients with Alzheimer's disease (AD)
- Participants with mild AD were treated with either placebo, 1 mg, 5 mg, or 15 mg of ABBV‐552 covering an estimated 35% to 80% SV2A receptor occupancy in a phase II randomized clinical trial
- Results failed to show efficacy over placebo as measured by ADAS‐Cog 14 at week 12
- ABBV‐552 was generally safe and well tolerated

## 1 BACKGROUND

Alzheimer's disease (AD), a progressive neurodegenerative condition characterized by the presence of amyloid beta (Aβ) plaques and tau pathologies, is the most common cause of dementia in elderly patients,^1^ often with mixed pathology.^2^ In 2023, more than 55 million people worldwide had dementia, with 60% to 70% of dementia cases attributed to AD.^3^ Current treatments are aimed at minimizing or slowing cognitive and functional declines associated with AD. Although initial symptoms may be mild, AD typically progresses to amnestic changes and cognitive deficits, including aphasia, apraxia, and agnosia. This is often accompanied by the emergence of neuropsychiatric symptoms such as confusion, agitation, depression, and, in some cases, hallucinations and psychosis, together leading to progressive loss of independence and a substantial increase in caregiver burden.

Currently, two classes of non‐disease‐modifying cognitive enhancers serve as symptomatic treatments for AD, including the acetylcholine inhibitors (AChEIs) donepezil, rivastigmine, and galantamine, and an *N*‐methyl‐d‐aspartate receptor antagonist, memantine.^4^ Although AChEIs, often used to treat patients with mild to moderate AD,^5^ have shown some efficacy in treating cognitive deficits, their efficacy lessens as cognitive deficits increase over time. For patients with moderate to severe AD, memantine is an option^5^, ^6^; however, not all patients demonstrate improvement. Brexpiprazole, a partial agonist of dopamine and serotonin receptors,^7^ has only recently been approved to treat agitation in patients with AD.^1^ Disease‐modifying treatments (DMTs) targeting Aβ accumulation and/or tau pathologies^5^, ^8^, ^9^, ^10^, ^11^, ^12^, ^13^, ^14^ are under investigation; two approved therapies that target Aβ accumulation are used both inside and outside the United States.^15^ While these DMTs slow the progression of the disease, they do not improve the symptoms of AD, making the development of more effective symptomatic treatment essential.

Synaptic vesicle glycoprotein 2A (SV2A), an integral membrane protein ubiquitously expressed in neuronal presynaptic vesicles, regulates calcium‐evoked vesicle fusion through its interaction with synaptotagmin,^16^ priming the exocytotic machinery for neurotransmitter release. In central nervous system diseases associated with impaired cognition, SV2A density is decreased,^17^, ^18^ including in patients with AD,^18^ leading to increasing use of SV2A ligands as biomarkers of pathology in AD.^19^

Given the critical role of SV2A in neuronal communication, targeting SV2A presents a promising therapeutic approach to treating AD. ABBV‐552, an orally bioavailable small molecule positive modulator of SV2A, binds SV2A with high affinity.^17^ ABBV‐552 demonstrates high selectivity for SV2A over other SV2 isoforms, SV2B and SV2C, and demonstrates procognitive activity in animal models of cognition.^16^, ^17^ We hypothesize that, by positively modulating the release of presynaptic vesicles, the efficiency of neurotransmission would increase, thereby compensating for loss of synapses and restoring neural connectivity in patients with mild AD.

To evaluate the safety of this compound, a recent phase I study of ABBV‐552 demonstrated that ascending single doses of up to 80 mg were generally safe and well tolerated in healthy male human subjects.^16^ The study demonstrated dose‐proportional pharmacokinetics over dosages ranging from 0.3 to 80 mg, with an observed terminal elimination half‐life of 34 to 50 h. The pharmacokinetic profile of ABBV‐552 is favorable and supports daily administration, with correlations between plasma concentration of ABBV‐552 and SV2A occupancy at doses between 1 and 20 mg demonstrated with positron emission tomography (PET).^16^ Based on these data, predicted ABBV‐552 receptor occupancy ranged from 35% to 80% in the dosing range of 1 to 15 mg that was selected for use in the current study, with animal studies supporting efficacy within this dosing range.^16^, ^17^

The purpose of this dose‐finding phase IIb study was to evaluate the safety and efficacy of ABBV‐552 once daily for the treatment of dementia in patients with clinically diagnosed mild AD, with the primary hypothesis that at least one dose of ABBV‐552 (i.e., 1, 5, or 15 mg) would be superior to placebo in improving cognitive function, as measured by the primary endpoint, change from baseline at week 12 in the 14‐item Alzheimer's Disease Assessment Scale‐Cognitive Subscale (ADAS‐Cog 14).

## 2 METHODS

### 2.1 Study design overview

This phase IIb, proof‐of‐concept, dose‐finding, multicenter, double‐blind, randomized, placebo‐controlled study (NCT05771428) was conducted at 53 sites in seven countries. Eligible participants were adults (males and females) between the ages of 50 and 90 years, inclusive, at time of consent who met criteria for probable AD according to the National Institute on Aging and the Alzheimer's Association 2011 criteria (2011 NIA‐AA), a Mini‐Mental State Examination (MMSE) score of 20 to 26, a Clinical Dementia Rating Scale (CDR) global score of 0.5 to 1, with a CDR memory score of 0.5 or more, and at least one CDR functional domain (community affairs, home and hobbies, or personal care) of 0.5 or more at the first screening visit. The MMSE range for inclusion was selected based on the hypothesis that it would be inclusive of participants who would most likely benefit from therapies that improve synaptic function while minimizing potential placebo responses. Eligible participants were required to have magnetic resonance imaging lacking evidence of alternative etiology for dementia other than AD, without another contributing cause of cognitive impairment or history of significant neurological disease. Eligible participants may have been naïve to AD symptomatic therapy, on a stable dose of AD therapy, or have discontinued any prior AD therapies at least 90 days before the first screening visit. Participants on anti‐amyloid therapies were excluded from this study to avoid potential factors that could confound our understanding of the safety and efficacy of ABBV‐552. Since ABBV‐552 is a symptomatic treatment with a mechanism that is not directed against amyloid or tau, we did not add PET/cerebrospinal fluid (CSF) as an inclusion criterion to minimize the burden of testing on patients. All eligible participants were also required to have a study partner (i.e., family member or caregiver) who spent sufficient time with the patient to provide information on the patient's functional and cognitive abilities. Patients who were unable to meet the inclusion criteria were excluded from this study.

The study consisted of a 30‐day screening period, a 12‐week, double‐blind treatment period, and a 30‐day safety follow‐up period. A 12‐week study duration was selected based on ABBV‐552's intended use as a symptomatic treatment for AD, given that other approved symptomatic treatments such as donepezil and rivastigmine demonstrated statistically significant treatment differences versus placebo at 3 and 12 weeks, respectively.^20^, ^21^ Since this was a dose‐finding study, four different doses were selected for study. To reduce the impact of practice effects from repeated administration of the ADAS‐Cog 14 and Cogstate Computerized Battery (CBB) assessments, participants were familiarized with these assessments during the screening period prior to the baseline assessment. Upon completion of all baseline assessments on day 1, participants received the first dose of study drug.

Before engaging in any study activities, all participants and study partners were required to provide written informed consent. All trial sites obtained approval for this study from an independent ethics committee or Institutional Review Board before initiating any study activities (Table S1). This study adhered to the tenets of the 1964 Declaration of Helsinki and its later amendments and was conducted in accordance with the International Conference on Harmonization Guidelines for Good Clinical Practice.

### 2.2 Blinding

After completing all baseline assessments at the first treatment visit, participants were randomized 1:1:1:1 to once daily placebo, ABBV‐552 1 mg, ABBV‐552 5 mg, or ABBV‐552 15 mg and stratified based on the presence or absence of a stable dose of symptomatic treatment for AD at baseline. All participants were assigned a unique identification number via interactive response technology at screening visit 1 according to the randomization schedule; this unique identifier was used throughout the study to encode the appropriate study drug to be dispensed at the participant's corresponding study visit. The participants, investigators, sponsor (apart from the sponsor's drug supply management team), and study site personnel remained blind to the treatment throughout the study. To maintain study anonymity, the ABBV‐552 and placebo tablets were identical in appearance.

**RESEARCH IN CONTEXT**

1. **Systematic review**: Although a limited number of studies using small molecule modulators of SV2A exist, a review of the literature using PubMed, meeting abstracts, and presentations demonstrated that very little is known about the potential benefits of SV2A modulation in patients with mild Alzheimer's disease (AD).
2. **Interpretation**: Preliminary studies in animals suggested a potential benefit of SV2A modulation in models of AD. This randomized, controlled clinical trial aimed to determine the efficacy of a small molecule positive SV2A modulator, ABBV‐552, for the treatment of AD. Although ABBV‐552 was found to be generally safe and well‐tolerated, results did not demonstrate efficacy over placebo at the dosages tested, as seen in the primary efficacy outcome of ADAS‐Cog 14 at week 12.
3. **Future directions**: Current results do not warrant further studies of ABBV‐552 in AD.

### 2.3 Efficacy measures

The primary efficacy endpoint was the change from baseline in ADAS‐Cog 14 at week 12 in the modified intent‐to‐treat (mITT) population, which included any participants who received at least one dose of study drug and had at least one post‐baseline ADAS‐Cog 14 score. ADAS‐Cog 14 was selected as the primary endpoint because this study sought to understand the effect of ABBV‐552 on cognition. Other efficacy endpoints include the Clinical Dementia Rating Scale–Sum of Boxes (CDR‐SB), the Neuropsychiatric Inventory (NPI), the MMSE, and scores from the Cogstate International Shopping List Test and the Cogstate Brief Battery (an assessment consisting of four neuropsychological tests: Detection, Identification, One Card Learning, and One Back), hereafter referred to in the aggregate as the Cogstate Computerized Battery (CCB), as well as composite scores from Alzheimer's Disease Composite Score (ADCOMS), which is a weighted composite score that combines components from the ADAS‐Cog 14, MMSE, and CDR‐SB.^22^

### 2.4 Pharmacokinetic (PK) measures

Blood samples to determine ABBV‐552 plasma concentrations were collected at baseline and weeks 1, 2, 6, and 12. A non‐linear mixed‐effects modeling approach was used to analyze ABBV‐552 plasma concentrations in mild AD, which was then used to update a previous population PK model of ABBV‐552 in healthy volunteers. A Gaussian regression model was used to relate ABBV‐552 exposure to the efficacy endpoints for both ADAS‐Cog 14 and CDR‐SB. Simulations were then performed using the exposure‐response model to predict the response for placebo and 1, 5, and 15 mg of ABBV‐552 after 12 weeks of daily administration.

### 2.5 Safety measures

Safety and demographic analyses were conducted on the safety population, which included all randomized participants who received at least one dose of study drug. Participants were classified according to the treatment they received most frequently, determined by the total number of days each treatment was administered. Safety evaluations included adverse event (AE) monitoring, weight, vital sign measurements, clinical laboratory testing (hematology, chemistry, and urinalysis), electrocardiogram (ECG) variables, and Columbia‐Suicide Severity Rating Scale (C‐SSRS) scores. AE severity was assessed according to the National Cancer Institute Common Terminology Criteria for Adverse Events (NCI CTCAE Version 4.0 or higher).

### 2.6 Statistical analysis

All efficacy analyses were conducted on the mITT analysis set; baseline was defined as the last non‐missing value prior to the first administration of study treatment. Sample size calculations were based on the assumption of a treatment difference between the ABBV‐552 and placebo groups of 2.85 in the ADAS‐Cog 14 change from baseline at week 12, with a pooled standard deviation (SD) of 5.7 (effect size 0.5), based on studies of symptomatic treatment of mild/moderate AD.^23^, ^24^, ^25^, ^26^ Assuming a 20% discontinuation rate (i.e., no post‐baseline data), 60 randomized participants per group were expected to provide ∼80% power to detect treatment differences in ADAS‐Cog 14 score at the two‐sided 10% significance level. The calculation was conducted using Cytel EAST version 6.5.3 (Cytel, Waltham, MA, USA).

Categorical variables were summarized with the number and percentage of participants; continuous variables were summarized with descriptive statistics (mean and SD). The primary endpoint, change from baseline in the ADAS‐Cog 14 score, was evaluated using a mixed‐effects model repeated measures (MMRM) analysis, including treatment group, visit, treatment‐by‐visit interaction, and the randomization stratum as fixed effects and the baseline ADAS‐Cog 14 score as a covariate. The unstructured covariance structure was used to estimate the within‐participant variance–covariance. Denominator degrees of freedom were evaluated with the Kenward–Roger method.^27^ The group mean treatment difference between each dose and placebo at week 12 was based on contrasts from this model. Missing efficacy data due to a patient's discontinuation from the study were assumed to be missing at random and were handled using a MMRM for the primary analysis. Efficacy data collected after a patient initiated, changed, or stopped treatment with an AD medication during the study were treated as missing and not included in the primary analysis. For endpoints measured at a single post‐baseline time point, the between‐group treatment summary was based on an analysis of covariance (ANCOVA), including treatment group, randomization stratum, and baseline for the respective parameter.

Change from baseline in ADAS‐Cog 14 and CDR‐SB at week 12 were also evaluated for the following subgroups: those on/not on symptomatic treatment for AD at baseline; sex; age < 65 years, ≥65 to <75 years, and ≥75 years; race and ethnicity; and amyloid probability score version 2 (APS2) status (APS2−, <48; APS2+, ≥48). The APS score was determined using the PrecivityAD2 blood test, a mass spectrometry‐based test that measures the percentage of phosphorylated tau protein and the Aβ42/40 ratio. Possible scores range from 0 to 100, with a score of 48 or higher indicating a higher probability of amyloid plaque accumulation in the brain.^28^, ^29^

The following additional efficacy endpoints were analyzed in the mITT population at week 12: change in the CCB, change in the MMSE total score, NPI change, and ADCOMS. Analysis of efficacy endpoints was based on a two‐sided *α* = 0.1 level. In the PK analyses, association of ABBV‐552 exposure and efficacy endpoints was evaluated based on *p* < 0.05. Analyses were performed with SAS version 9.4 (SAS Institute Inc., Cary, NC, USA) or later.

Sensitivity analyses of the primary endpoint included imputation using the copy‐reference approach^30^ and alternate handling of intercurrent events. The copy‐reference approach assessed the robustness of the MMRM analysis to any possible violation of the missing‐at‐random assumption in the primary analysis. Two additional sensitivity analyses of the primary endpoint were evaluated through alternative handling of intercurrent events combined with the same MMRM specified in the primary analysis. In the first additional sensitivity analysis, efficacy data collected after a start, stop, or change of dose of AD medication during the study was evaluated. In the second sensitivity analysis, efficacy data collected after a participant discontinued study treatment was excluded from the primary analysis.

### 2.7 Data availability

AbbVie is committed to responsible data sharing regarding the clinical trials we sponsor. This includes access to anonymized, individual, and trial‐level data (analysis data sets), as well as other information (e.g., protocols, clinical study reports, synopses, or statistical analysis plans), as long as the trials are not part of an ongoing or planned regulatory submission.

These clinical trial data can be requested by any qualified researchers who engage in rigorous, independent, scientific research and will be provided following review and approval of a research proposal, Statistical Analysis Plan, and execution of a data use agreement (DUA). Data requests can be submitted at any time after approval in the United States and Europe and after acceptance of this manuscript for publication. The data will be accessible for 12 months, with possible extensions considered. For more information on the process or to submit a request, visit the following link: https://vivli.org/ourmember/abbvie/ then select “Home.”

## 3 RESULTS

### 3.1 Demographic, baseline characteristics, and subject dispositions

**FIGURE 1:**
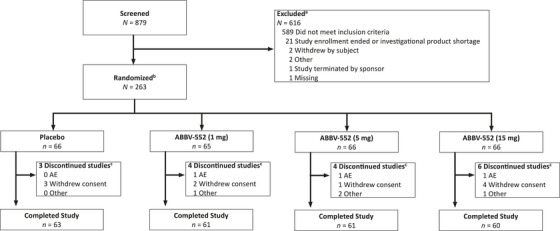
Patient disposition. ^a^Patients may have more than one reason for exclusion. ^b^Includes all randomized participants who had ≥1 dose of study drug in the intent‐to‐treat population. ^c^Primary reasons for study discontinuation. AE = adverse event.

Enrollment began in April of 2023 and ended in August of 2024. Of the 879 individuals screened, 263 were randomized to receive one of three doses of ABBV‐552: 1 mg (*n* = 65), 5 mg (*n* = 66), 15 mg (*n* = 66), or placebo (*n* = 66) (Figure 1). Of the 263 randomized participants, 262 took at least one dose of the study drug. A total of 245 (93.2%) participants completed the study, with an overall discontinuation rate of 6.5%. A similar proportion of participants across treatment groups discontinued the study (ABBV‐552 1 mg [*n* = 4, 6.2%], ABBV‐552 5 mg [*n* = 4, 6.1%], ABBV‐552 15 mg [*n* = 6, 9.1%], and placebo [*n* = 3, 4.6%]). Withdrawal from treatment by subject was the main reason for study discontinuation.

Baseline demographics were generally balanced across treatment arms (Table 1). The overall safety population's mean age was 73.1 years, with 55.7% female; 78.8% of the participants were White, 10.0% were Asian, and 10.4% were Black or African American. The mean (SD) time from symptom onset and time from AD disease diagnosis were 4.435 (3.43) years and 1.93 (2.93) years, respectively. A total of 115/255 (45.1%) participants in the mITT population were on symptomatic treatment (i.e., cholinesterase inhibitors and/or memantine) for AD. Of this group, 252/255 (98.8%) had no change in treatment over the course of this study.

**TABLE 1:** Demographics and baseline characteristics (safety population).

	Placebo (*N* = 66)	ABBV‐552, 1 mg (*N* = 65)	ABBV‐552, 5 mg (*N* = 65)	ABBV‐552, 15 mg (*N* = 66)	Total (*N* = 196)	Overall (*N* = 262)
Sex, *n* (%)						
Female	30 (45.5)	40 (61.5)	43 (66.2)	33 (50.0)	116 (59.2)	146 (55.7)
Ethnicity, *n* (%)						
Hispanic or Latino	20 (30.3)	25 (38.5)	27 (41.5)	22 (33.3)	74 (37.8)	94 (35.9)
Race, *n* (%)						
American Indian or Alaska Native	0	1 (1.6)	0	0	1 (0.5)	1 (0.4)
Asian	6 (9.1)	3 (4.7)	7 (10.8)	10 (15.4)	20 (10.3)	26 (10.0)
Black or African American	8 (12.1)	5 (7.8)	7 (10.8)	7 (10.8)	19 (9.8)	27 (10.4)
White	52 (78.8)	55 (85.9)	51 (78.5)	47 (72.3)	153 (78.9)	205 (78.8)
Multiple	0	0	0	1 (1.5)	1 (0.5)	1 (0.4)
Missing	0	1	0	1	2	2
Age in years, *n* (%)						
<65	13 (19.7)	6 (9.2)	7 (10.8)	10 (15.2)	23 (11.7)	36 (13.7)
65 to 74	20 (30.3)	31 (47.7)	25 (38.5)	29 (43.9)	85 (43.4)	105 (40.1)
75 to 84	28 (42.4)	25 (38.5)	30 (46.2)	23 (34.8)	78 (39.8)	106 (40.5)
≥85	5 (7.6)	3 (4.6)	3 (4.6)	4 (6.1)	10 (5.1)	15 (5.7)
Body mass index in kg/m^2^, *n* (%)						
<25	17 (25.8)	23 (35.4)	22 (34.4)	21 (31.8)	66 (33.8)	83 (31.8)
≥25	49 (74.2)	42 (64.6)	42 (65.6)	45 (68.2)	129 (66.2)	178 (68.2)
Missing	0	0	1	0	1	1
Likelihood of brain amyloid pathology, *n* (%)						
APS2− (≤48)	32 (51.6)	26 (43.3)	32 (54.2)	29 (46.0)	87 (47.8)	119 (48.8)
APS2+ (>48)	30 (48.4)	34 (56.7)	27 (45.8)	34 (54.0)	95 (52.2)	125 (51.2)
Missing	4	5	6	3	14	18
Time since symptom onset in years, mean (SD)^a^	4.8 (3.3)	3.7 (2.7)	4.6 (3.7)	4.5 (3.8)	4.3 (3.5)	4.4 (3.4)
Time since diagnosis in years, mean (SD)^b^	1.6 (2.4)	1.7 (2.2)	2.3 (3.4)	2.1 (3.5)	2.0 (3.1)	1.9 (2.9)
AD symptom treatment at baseline, *n* (%)						
Never treated	39 (59.1)	33 (50.8)	34 (52.3)	34 (51.5)	101 (51.5)	140 (53.4)
Previously treated and ongoing	27 (40.9)	30 (46.2)	30 (46.2)	31 (47.0)	91 (46.4)	118 (45.0)
Previously treated and stopped	0	2 (3.1)	1 (1.5)	1 (1.5)	4 (2.0)	4 (1.5)
Baseline ADAS‐Cog 14 and CDR‐SB,^c^ *n* (%)						
Baseline ADAS‐Cog 14, mean (SD)^d^	24.1 (8.3)	23.0 (8.6)	23.2 (7.8)	23.8 (8.3)	n/a	n/a
Baseline CDR‐SB, mean (SD)^e^	3.6 (1.3)	3.5 (1.2)	3.6 (1.4)	3.8 (1.5)	n/a	n/a

*Note*: Safety population includes all randomized patients who had ≥1 dose of study drug. Percentages calculated on non‐missing values. Participants self‐identified sex as male or female; race and ethnicity as Hispanic or Latino, American Indian or Alaska Native, Asian, Black or African American, White, or more than one race.

Abbreviations: AD, Alzheimer's disease; ADAS‐Cog 14, Alzheimer's Disease Assessment Scale (14‐Item) Cognitive Subscale; APS2, Amyloid Probability Score 2; CDR‐SB, Clinical Dementia Rating Scale–Sum of Boxes; n/a, not analyzed; SD, standard deviation.

^a^Placebo, *n* = 61; ABBV‐552 1.0 mg, *n* = 64; ABBV‐552 5.0 mg, *n* = 64; ABBV‐552 15.0 mg, *n* = 64; total, *n* = 192; overall, *n* = 253.

^b^Placebo, *n* = 65; ABBV‐552 1.0 mg, *n* = 64; ABBV‐552 5.0 mg, *n* = 65; ABBV‐552 15.0 mg, *n* = 65; total, *n* = 194; overall, *n* = 259.

^c^In modified intent‐to‐treat set, which includes participants who received at least one dose of study drug and had at least one post‐baseline ADAS‐Cog 14 assessment score.

^d^Placebo, *n* = 64; ABBV‐552 1.0 mg, *n* = 63; ABBV‐552 5.0 mg, *n* = 64; ABBV‐552 15.0 mg, *n* = 63.

^e^Placebo, *n* = 65; ABBV‐552 1.0 mg, *n* = 63; ABBV‐552 5.0 mg, *n* = 64; ABBV‐552 15.0 mg, *n* = 63.

Disease characteristics at baseline (Table 1) were relatively similar among treatment groups, including baseline total mean ADAS‐Cog 14 score, CDR‐SB scores, and the APS2 score as a predictive blood biomarker for brain amyloidosis.^28^, ^29^ The APS2 score is a composite measure based on the Aβ42/40 ratio and the ratio of phosphorylated to non‐phosphorylated tau‐217, providing a more comprehensive measure of brain amyloidosis than either measure alone.^28^, ^29^ The mean (SD) baseline total ADAS‐Cog 14 scores were as follows: placebo, 24.10 (8.26); ABBV‐552 1 mg, 22.99 (8.57); ABBV‐552 5 mg, 23.24 (7.78); and ABBV‐552 15 mg, 23.82 (8.27). The mean (SD) baseline CDR‐SB scores were as follows: placebo, 3.60 (1.28); ABBV‐552 1 mg, 3.52 (1.20); ABBV‐552 5 mg, 3.63 (1.35); and ABBV‐552 15 mg, 3.75 (1.45).

**TABLE 2:** MMRM analysis of ADAS‐Cog 14 total score mean change at week 12 in the mITT^a^ population.

				Model‐based between‐group difference compared with placebo (*N* = 254)^b^		
Treatment arm	*N* c	Baseline mean (SD)	Week 12 (SD)	LS mean (SE)	95% CI	*p* value
Placebo	58	23.71 (8.238)	22.32 (8.940)	–	–	–
ABBV‐552 1 mg	56	22.20 (8.731)	20.79 (9.638)	−0.02 (0.939)	[−1.87, 1.83]	0.9819
ABBV‐552 5 mg	58	22.93 (7.951)	21.23 (9.314)	−0.42 (0.931)	[−2.25, 1.42]	0.6545
ABBV‐552 15 mg	59	24.10 (8.339)	23.01 (9.793)	0.25 (0.928)	[−1.58, 2.08]	0.7860

*Note*: MMRM analysis includes treatment, visit, treatment‐by‐visit interaction, and whether or not the subject was taking symptomatic treatment for Alzheimer's disease at baseline as fixed factors and baseline value as covariate. Denominator degrees of freedom computed using the Kenward–Roger method. Unstructured covariance structure is used. A lower score on ADAS‐Cog 14 indicates better performance.

Abbreviations: CI, confidence interval; MMRM, mixed‐effect model with repeated measures; SD, standard deviation; SE, standard error.

^a^Includes all randomized participants who received at least one capsule of study drug and have at least one post‐baseline ADAS‐Cog 14 score.

^b^Number of unique subjects contributing to the model estimates (not visit‐specific).

^c^Number of subjects with observed measurements at both baseline and at 12 weeks.

### 3.2 Outcomes

#### 3.2.1 ADAS‐Cog 14

**FIGURE 2:**
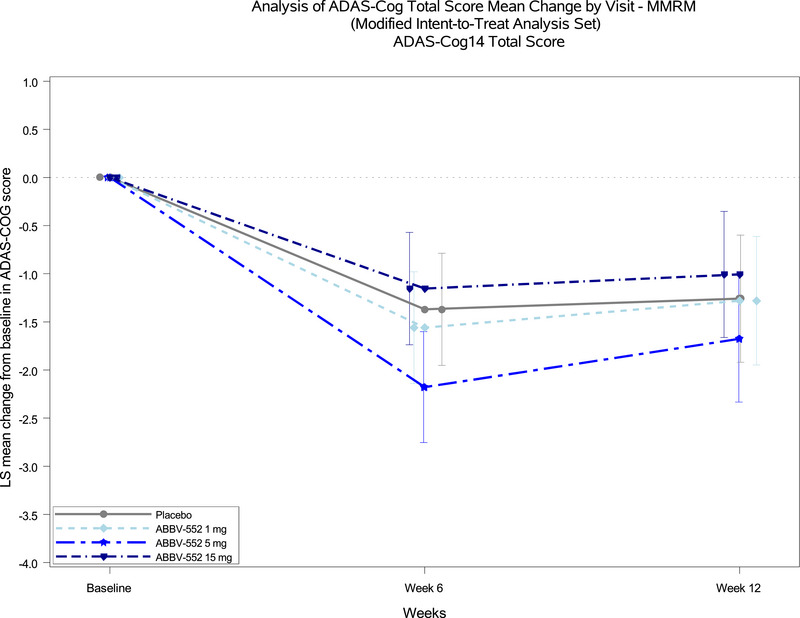
Analysis of 14‐item Alzheimer's Disease Assessment Scale‐Cognitive Subscale (ADAS‐Cog 14) total score mean change by visit using a mixed model for repeated measures in the modified intent‐to‐treat (mITT) analysis set. Shown is the least‐squares mean change from baseline in ADAS‐Cog 14 total score by visit in the mITT population, including all randomized participants who received at least one capsule of study drug and had at least one post‐baseline ADAS‐Cog 14 score.

The mean change in ADAS‐Cog 14 total scores by visit was analyzed using a MMRM approach in the mITT analysis set. Among participants who received ABBV‐552 1, 5, and 15 mg and placebo, the overall cognitive performance did not differ on the ADAS‐Cog 14 between ABBV‐552 treatment groups and placebo (Table 2). Results of the sensitivity analysis, conducted by including efficacy data collected after any start, stop, or change in dose of AD medication and excluding data after treatment discontinuation, were consistent with the primary analysis. The ADAS‐Cog 14 least‐squares (LS) mean change from baseline in total score at weeks 6 and 12 is shown in Figure 2. Subgroup analyses of ADAS‐Cog 14 total score mean change from baseline showed no treatment difference between any of the ABBV‐552 treatment arms versus placebo for any of the subgroups analyzed when stratified by age, sex, concurrent symptomatic AD treatment, race and ethnicity, or APS2 status.

#### 3.2.2 CDR‐SB

At week 12, the mean change in CDR‐SB scores was comparable between all ABBV‐552 treatment arms versus placebo, as presented in Table 3, except for some changes in the 5‐mg arm. At week 12, participants receiving 5 mg ABBV‐552 showed a LS mean treatment difference versus placebo in the CDR‐SB score of −0.42 (SD 0.214), with a nominal *p* value of 0.0518, unadjusted for multiplicity, smaller than 0.10 alpha level. The 95% confidence interval (CI) for this change was −0.84 to 0.00. Subgroup analysis of CDR‐SB mean change at week 12 for all treatment arms was comparable to placebo, except for nominal differences in the 5‐mg arm versus placebo for the following subgroups: participants who were not on symptomatic treatment for AD, female, age ≤ 65, between age 65 and <75, White, or APS2 negative (APS2−) (Table S2).

**TABLE 3:** ANCOVA analysis of CDR‐SB mean score change at week 12 (mITT^a^ population).

				Model‐based (*N* = 241)^b^ change from baseline, group difference results			
Treatment arm	*N* ^c^	Baseline mean (SD)	Week 12 (SD)	LS mean (SE)	95% CI	*P* value	Effect size
Placebo	61	3.52 (1.231)	3.55 (1.583)	–	–	–	–
ABBV‐552 1 mg	60	3.50 (1.211)	3.46 (1.781)	−0.11 (0.214)	[−0.53, 0.32]	0.6223	0.09
ABBV‐552 5 mg	61	3.59 (1.312)	3.22 (1.532)	−0.42 (0.214)	[−0.84, 0.00]	0.0518	0.35
ABBV‐552 15 mg	59	3.74 (1.478)	3.65 (1.740)	−0.11 (0.216)	[−0.54, 0.31]	0.6016	0.10

*Note*: ANCOVA includes treatment group and stratification factor as main effects and the baseline as a covariate. Effect size is calculated as model‐based group difference divided by pooled SD.

Abbreviations: ANCOVA, analysis of covariance; CDR‐SB, Clinical Dementia Rating Scale–Sum of Boxes; CI, confidence interval; LS, least squares; SD, standard deviation; SE, standard error.

^a^Includes all randomized participants who received at least one capsule of study drug and had at least one post‐baseline ADAS‐Cog 14 score.

^b^Number of unique subjects contributing to the model estimates (not visit‐specific).

^c^Number of subjects with observed measurements at both baseline and at 12 weeks.

#### 3.2.3 Pharmacokinetics

Exposure–response analyses showed no meaningful association between the average plasma concentration of ABBV‐552 and either ADAS‐Cog 14 total score at week 12 or CDR‐SB at week 12 (Table S3).

#### 3.2.4 Cogstate Computerized Battery

No meaningful treatment differences were observed between any of the ABBV‐552 treatment arms versus placebo in the CCB composite assessments (Global, Attention Domain, Learning/Working Memory, and Episodic Memory Domain) at week 12 (Table 4). The Cogstate Global Composite assessment includes the Detection Test, Identification Test, One Card Learning, One Back Test, and International Shopping List Test (both immediate recall and delayed recall). The Cogstate Attention Domain Composite includes the Detection Test and the Identification Test. The Cogstate Learning/Working Memory Composite includes the One Card Learning and One Back tests. The Cogstate Episodic Domain Composite includes the International Shopping List test (both immediate and delayed recall).

**TABLE 4:** MMRM analysis Cogstate Computerized Battery Global Composite Score at week 12 (mITT^a^ population).

				Model‐based change from baseline, group difference results versus placebo			
Treatment arm	*N* ^b^	Baseline mean (SD)	Week 12 (SD)	LS mean (SE)	95% CI	*P* value	Effect size
**Global Composite Score**							
Placebo	59	−1.53 (0.683)	−1.31 (0.652)	(*N* = 253)^c^			
ABBV‐552 1 mg	59	−1.28 (0.776)	−1.27 (0.775)	−0.19 (0.090)	[−0.36, −0.01]	0.0399^*^	−0.38
ABBV‐552 5 mg	59	−1.34 (0.765)	−1.20 (0.928)	−0.06 (0.090)	[−0.24, 0.12]	0.4946	−0.13
ABBV‐552 15 mg	57	−1.47 (0.843)	−1.42 (0.784)	−0.17 (0.091)	[−0.34, 0.01]	0.0690	−0.34
**Attention Domain Composite Score**							
Placebo	62	−1.35 (1.044)	−1.18 (0.961)	(*N* = 253)^c^			
ABBV‐552 1 mg	58	−0.99 (1.025)	−1.13 (1.074)	−0.24 (0.140)	[−0.52, 0.03]	0.0845	−0.32
ABBV‐552 5 mg	57	−1.02 (1.038)	−1.15 (1.108)	−0.20 (0.140)	[−0.47, 0.08]	0.1649	−0.26
ABBV‐552 15 mg	57	−1.26 (1.380)	−1.21 (1.248)	−0.12 (0.140)	[−0.40, 0.16]	0.3933	−0.16
**Learning/Working Memory Domain Composite Score**							
Placebo	56	−1.27 (1.192)	−0.97 (1.127)	(*N* = 246)^c^			
ABBV‐552 1 mg	56	−1.21 (1.134)	−1.16 (1.173)	−0.25 (0.156)	[−0.56, 0.05]	0.1048	−0.31
ABBV‐552 5 mg	57	−1.26 (1.031)	−0.97 (1.246)	−0.04 (0.155)	[−0.34, 0.27]	0.8135	−0.04
ABBV‐552 15 mg	56	−1.02 (1.087)	−1.01 (1.068)	−0.24 (0.156)	[−0.55, 0.06]	0.1200	−0.29
**Episodic Memory Domain Composite Score**							
Placebo	56	−1.89 (0.954)	−1.79 (1.031)	(*N* = 249)^c^			
ABBV‐552 1 mg	59	−1.63 (1.036)	−1.50 (1.063)	0.07 (0.135)	[−0.20, 0.33]	0.6212	0.09
ABBV‐552 5 mg	59	−1.68 (1.097)	−1.44 (1.250)	0.17 (0.135)	[−0.09, 0.44]	0.2019	0.24
ABBV‐552 15 mg	56	−2.00 (0.971)	−1.95 (1.004)	−0.06 (0.136)	[−0.33, 0.21]	0.6603	−0.08

*Note*: MMRM analysis includes treatment, visit, treatment‐by‐visit interaction, and whether or not the subject was taking symptomatic treatment for Alzheimer's disease at baseline as fixed factors and baseline value as covariate. Denominator degrees of freedom computed using the Kenward–Roger method. Unstructured covariance structure is used. Effect size is calculated as model‐based group difference divided by pooled SD (Cohen's *d*). Higher scores indicate better performance.

Abbreviations: CI, confidence interval; LS, least squares; MMRM, mixed‐effect model with repeated measures; SD, standard deviation; SE, standard error.

^a^Includes all randomized participants who received at least one capsule of study drug and had at least one post‐baseline ADAS‐Cog 14 score.

^b^Number of subjects with observed measurements at both baseline and at 12 weeks

^c^Number of unique subjects contributing to the model estimates (not visit‐specific).

*p ≤ 0.05; **p ≤ 0.01; ***p ≤ 0.001.

#### 3.2.5 MMSE, NPI, and ADCOMS

No meaningful treatment differences were observed between any of the ABBV‐552 treatment arms versus placebo in the NPI and MMSE analyses at week 12. Analysis of ADCOMS at week 12 revealed a LS mean change (SE) from baseline for the ABBV‐552 5 mg treatment arm versus placebo of −0.05 (0.021), 95% CI (−0.09 to −0.01), nominal *p* value = 0.0268.

### 3.3 Safety

Overall, ABBV‐552 was generally well tolerated, and no safety concerns were identified. The incidence of treatment‐emergent adverse events (TEAEs), serious adverse events (SAEs), and TEAEs leading to study drug discontinuation were comparable between ABBV‐552 and placebo groups (Table 5).

**TABLE 5:** Overview of treatment‐emergent adverse events (TEAEs) in safety population.

		ABBV‐552			
AEs, *n* (%)	Placebo (*n* = 66)	1 mg (*n* = 65)	5 mg (*n* = 65)	15 mg (*n* = 66)	Total (*n* = 196)
Any treatment‐emergent AE	28 (42.4)	32 (49.2)	29 (44.6)	34 (51.5)	95 (48.5)
Any treatment‐emergent AE related to study treatment^a^	8 (12.1)	8 (12.3)	15 (23.1)	14 (21.2)	37 (18.9)
Any treatment‐emergent AE Grade 3 or higher	2 (3.0)	4 (6.2)	2 (3.1)	2 (3.0)	8 (4.1)
Any serious treatment‐emergent AE	3 (4.5)	3 (4.6)	2 (3.1)	2 (3.0)	7 (3.6)
Any treatment‐emergent AE leading to discontinuation of study treatment	0	1 (1.5)	1 (1.5)	1 (1.5)	3 (1.5)
Any treatment‐emergent AE leading to death	0	0	1 (1.5)	0	1 (0.5)
All deaths	0	0	1 (1.5)	0	1 (0.5)
Most common AEs by preferred term,^b^ *n* (%)					
Dizziness	3 (4.5)	2 (3.1)	4 (6.2)	8 (12.1)	14 (7.1)
Fatigue	2 (3.0)	3 (4.6)	2 (3.1)	7 (10.6)	12 (6.1)
Upper respiratory tract infection	1 (1.5)	4 (6.2)	2 (3.1)	3 (4.5)	9 (4.6)
Diarrhea	0	1 (1.5)	5 (7.7)	2 (3.0)	8 (4.1)
Headache	2 (3.0)	2 (3.1)	4 (6.2)	2 (3.0)	8 (4.1)
Nasopharyngitis	2 (3.0)	2 (3.1)	1 (1.5)	4 (6.1)	7 (3.6)
Fall	5 (7.6)	2 (3.1)	2 (3.1)	2 (3.0)	6 (3.1)
Urinary tract infection	3 (4.5)	6 (9.2)	0	0	6 (3.1)
Somnolence	0	0	4 (6.2)	1 (1.5)	5 (2.6)

*Note*: Safety population includes all randomized participants who had ≥1 dose of study drug. Subjects are counted once in each row within a column, regardless of the number of events they may have had.

Abbreviation: AE, adverse event.

^a^According to investigator.

^b^Occurring in ≥5.0% of participants in any treatment group, listed by primary Medical Dictionary for Regulatory Activities v23.1 preferred term.

In total, 95 of 196 participants (48.5%) receiving any dose of ABBV‐552 experienced TEAEs, compared to 28 of 66 participants (42.4%) in the placebo group (Table 5). The majority of AEs reported during the treatment period were non‐serious and Grade 1 or 2 in severity. Seven participants (3.6%) had SAEs among all ABBV‐552 treatment groups, compared to three participants (4.5%) in the placebo group. No safety trends were identified for SAEs. One participant in the ABBV‐552 5 mg group had an SAE of a road traffic accident leading to death, which was assessed as not related to the study drug.

Three participants (1.5%) had at least one TEAE leading to study treatment discontinuation in any of the ABBV‐552 treatment arms, while none were reported in the placebo group. All events leading to study drug discontinuation were non‐serious.

The most common TEAEs reported by 5% or more of participants in the ABBV‐552 treatment groups were dizziness and fatigue (Table 5). No safety concerns were identified based on the review of laboratory results, vital signs, and ECG results, and there was no evidence of increased suicidality based on C‐SSRS (data not shown).

## 4 DISCUSSION

In this phase IIb trial evaluating ABBV‐552 in patients with mild AD, there was no meaningful difference between ABBV‐552 and placebo in the primary clinical outcome, change from baseline in ADAS‐Cog 14 at week 12. The results of sensitivity analyses and exploratory clinical outcomes were consistent with the results of the primary clinical outcome. Further, the lack of statistically significant associations between ABBV‐552 exposures and change in ADAS‐Cog 14 or CDR‐SB score at week 12 also supported the results from the primary analysis.

The failure to demonstrate a significant treatment difference in efficacy endpoints in the current trial underscores the challenges of treating AD, given the diversity of clinical manifestations and neuropathological features that contribute to AD, including Aβ plaques, neurofibrillary tangles, synaptic and neuronal loss, and neuroinflammation. Altogether, the heterogeneity of AD contributes to baseline variation that may have hampered detection of subtle changes from baseline after treatment. There was a slight numerical trend of improvement across all groups, including placebo, despite participants' completion of several ADAS‐Cog 14 assessments to minimize any possible practice effects prior to receipt of study drug. The improvements across all treatment groups noted here may be attributed to placebo effects; studies suggest that placebo effects in symptomatic AD trials peak between 6 and 12 weeks.^31^ A trial of longer duration would be expected to help minimize placebo effects as well as extend test–retest intervals that could further minimize any practice effects, as would the use of alternate test forms or modeling practice effects as a covariate. In this study, although participants engaged in practice assessments during the screening period to help minimize such effects, their influence on these negative findings cannot be ruled out.

While all enrolled participants were believed to have mild AD based on clinical assessments at screening, roughly half were found to be APS2+, which may have limited the ability to detect treatment changes in patients with amyloid pathology. In this study, biomarkers were not used as part of the eligibility criteria; participants were selected based on clinical assessments as per the 2011 NIA‐AA clinical criteria for AD diagnosis. The proportion of those found to be APS2− in the current study underscores the importance of biomarkers in confirming AD pathology prior to randomization.

The observed preponderance of female participants in our study aligns with established epidemiological data that indicates that AD disproportionately affects females^32^; the demographic characteristics demonstrated adequate diversity of races enrolled in this phase II trial.

The tolerability of ABBV‐552, along with the short study duration, likely contributed to the low discontinuation rate of 6.5%. The safety profile of ABBV‐552 was consistent with what was observed in earlier phase I studies in healthy volunteers.^16^ Dizziness and fatigue were the most commonly reported TEAEs (7.1% and 6.1%, respectively), which was not unexpected, given results in early ABBV‐552 studies with healthy volunteers, albeit more commonly at doses higher than those used in the current study.^16^ In this study, all events of dizziness and fatigue were non‐serious and mild to moderate in severity; most resolved without any intervention.

There was one death involving an automobile accident that the investigator considered related to the study drug. However, upon further investigation, the sponsor's causality assessment was that the accident was unlikely to be related to ABBV‐552, as the participant had a significant past medical history, including multiple vascular risk factors, which are potential confounders.

The current trial is the first multicenter randomized phase II clinical trial to explore the use of ABBV‐552 for patients with mild AD. Although positive modulation of SV2A is a novel approach for the treatment of AD, the current 12‐week trial failed to show a significant difference from placebo; a study duration exceeding 12 weeks may be necessary to reliably identify therapeutic benefits conferred by ABBV‐552,^33^ especially if those effects are modest. Outcome measures such as those used in the current study may lack the sensitivity to detect subtle functional differences in a population with mild cognitive impairment.^34^ The development of specific and sensitive biomarkers for AD^35^ and composite cognitive and functional measures may better allow detection of treatment‐related changes in clinical trials involving patients with early AD.^36^

### 4.1 Limitations

This study was limited in terms of sample size and study duration, which might have affected the detection of subtle treatment effects. We cannot rule out that these negative findings may be due, at least in part, to the insensitive nature of the endpoints, which may not reflect subtle changes that may have resulted from treatment with ABBV‐552, rather than a true lack of efficacy. This trial was designed as a dose‐ranging, proof‐of‐concept study and was not powered to demonstrate superiority of any one dose over placebo. Another limitation of this study is based on the fact that significant synaptic loss and neurodegeneration in AD occur before symptoms are evident^37^; thus, participants with mild AD may already have had disease progression such that synaptic loss was beyond the point at which ABBV‐552 could have made an impact. While magnetic resonance imaging scans were used to rule out alternate etiology of dementia, the use of the 2011‐AA clinical criteria for AD diagnosis without biomarker confirmation as part of the inclusion criteria may have resulted in a heterogeneous cohort, for example, inclusion of participants with non‐AD pathologies such as dementia with Lewy bodies. In this study, APS2 was used as a proxy for amyloid disease, rather than using amyloid PET/CSF to determine amyloid status. With new blood‐based biomarkers recently approved by the US Food and Drug Administration,^38^ future studies should leverage these sensitive biomarkers predictive of amyloid load as an inclusion criterion without concern for patient/site burden, potentially identifying subgroups that could benefit from this treatment.

## 5 CONCLUSION

Although ABBV‐552 was generally safe and well tolerated in participants with mild AD, ABBV‐552 1, 5, or 15 mg did not demonstrate meaningful improvement over placebo in the treatment of mild AD, as measured by a 2‐ to 3‐point improvement in the primary efficacy endpoint of ADAS‐Cog 14 at week 12. An exposure–response analysis also showed no statistically significant association between ABBV‐552 exposure and ADAS‐Cog 14 score improvement at week 12, confirming the primary analysis. Although ABBV‐552 showed promise in terms of safety, further studies are required to explore its potential to improve cognitive function.

## AUTHOR CONTRIBUTIONS

Shau Yu Lynch contributed to the research project conception and design, data acquisition, data interpretation, drafting, review and critique of the manuscript, and approval of the final manuscript draft submitted for publication. Jia Jia contributed to the statistical analysis, data interpretation, drafting, review, and critique of the manuscript, and approval of the final manuscript draft submitted for publication. Nick Miles contributed to the data interpretation, drafting, review and critique of the manuscript, and approval of the final manuscript draft submitted for publication. Joey Boiser contributed to the research project conception and design, data acquisition, data interpretation, drafting, review and critique of the manuscript, and approval of the final manuscript draft submitted for publication. Derek L. Buhl contributed to the data interpretation, drafting, review and critique of the manuscript, and approval of the final manuscript draft submitted for publication. Ole Graff contributed to the research project conception and design, data interpretation, drafting, review, and critique of the manuscript, and approval of the final manuscript draft submitted for publication. Cindy Zadikoff contributed to the research project conception and design, data acquisition, data interpretation, drafting, review and critique of the manuscript, and approval of the final manuscript draft submitted for publication. All authors agree to be accountable for all aspects of the work, ensuring the accuracy and integrity of the publication.

## CONFLICT OF INTEREST STATEMENT

Shau Yu Lynch, Jia Jia, Nick Miles, Joey Boiser, Derek L. Buhl, Ole Graff, and Cindy Zadikoff are employees of AbbVie Inc. and may hold AbbVie stock and/or stock options. AbbVie Inc. funded the research for this study and participated in the study design, study research, collection, analysis, and interpretation of data, as well as writing, reviewing, and approving this manuscript for publication. All authors had access to the data and participated in document development, review, approval, and the decision to submit this manuscript for publication. No honoraria or payments were made for authorship. Author disclosures are available in the supporting information.

## CONSENT STATEMENT

Before engaging in any study activities, all participants and study partners provided written informed consent.

## Supporting information



Supporting Information

Supporting Information
