# Supplementary material for: ABBV‐552 in patients with mild Alzheimer's disease: a randomized phase IIb trial
Source: Alzheimers Dement. 2025 Dec 26;21(12):e70994. doi: 10.1002/alz.70994 (PMC12741937; doi:10.1002/alz.70994)
Supplement: Supplementary file 1 — Supporting Information [file ALZ-21-e70994-s002.docx]

**Supplementary Table 1.** Names of Institutional Review Boards/Ethics Committees approving this study.

Advarra, Columbia, Maryland, USA

University of Kentucky Office of Research Integrity Medical IRB, Lexington, Kentucky, USA

CEIm de la Communidad Foral de Navarra, Pamplona, Navarra, Spain

St Vincent’s Hospital HREC, Darlinghurst, New South Wales, Australia

South Central – Berkshire B Research Ethics Committee, London, UK

Southern Health and Disability Ethics Committee, Wellington, Wellington, New Zealand

Hizen Psychiatric Center Institutional Review Board, Kanzaki-gun, Saga, Japan

Tokushukai Group Institutional Review Board, Chiyoda-ku, Tokyo, Japan

Oita University Hospital Institutional Review Board, Yufu-shi, Oita, Japan

Ethik-Kommission der Medizinischen Fakultaet der Christian-Albrechts Universitaet zu Kiel, Berlin, Berlin, Germany

Nara Medical University Hospital Institutional Review Board, Kashihara-shi, Nara, Japan

Juntendo University Hospital Institutional Review Board, Bunkyo-ku, Tokyo, Japan

NHO Niigata National Hospital Institutional Review Board, Kashiwazaki-shi, Niigata, Japan

Teikyo University Hospital, Mizonokuchi Institutional Review Board, Kawasaki-shi, Kanagawa, Japan

National Hospital Organization Hiroshimanishi Medical Center Institutional Review Board, Otake-shi, Hiroshima, Japan

| **Supplementary Table 2. Subgroup ANCOVA Analysis of CDR-SB Mean Change at Week 12 in the mITT^a^ Population** | | | | | | |
| --- | --- | --- | --- | --- | --- | --- |
|  |  |  |  | **Between-Group Difference Compared With Placebo (N=254)** | | |
| **Treatment Arm** | **N** | **Baseline Mean (SD)** | **Week 12 (SD)** | **LS Mean (SE)** | **95% CI** | **Nominal *P* value** |
| **Currently on Symptomatic Treatment for AD** | 111 |  |  |  |  |  |
| Placebo | 25 | 3.84 (1.21) | 4.00 (1.51) |  |  |  |
| ABBV-552 1 mg | 29 | 3.78 (1.37) | 3.86 (1.98) | -0.09 (0.34) | [-0.76, 0.58] | 0.798 |
| ABBV-552 5 mg | 29 | 3.47 (1.01) | 3.59 (1.32) | -0.12 (0.34) | [-0.79, 0.56] | 0.735 |
| ABBV-552 15 mg | 28 | 4.11 (1.61) | 4.00 (1.58) | -0.21 (0.34) | [-0.89, 0.47] | 0.535 |
| **Not Currently on Symptomatic Treatment for AD** | 130 |  |  |  |  |  |
| Placebo | 36 | 3.29 (1.21) | 3.24 (1.57) |  |  |  |
| ABBV-552 1 mg | 31 | 3.24 (1.00) | 3.08 (1.51) | -0.11 (0.27) | [-0.65, 0.43] | 0.693 |
| ABBV-552 5 mg | 32 | 3.70 (1.54) | 2.89 (1.65) | -0.74 (0.27) | [-1.28, -0.20] | **0.008** |
| ABBV-552 15 mg | 31 | 3.40 (1.28) | 3.34 (1.84) | 0.00 (0.27) | [-0.54, 0.54] | 0.988 |
| **Male** | 107 |  |  |  |  |  |
| Placebo | 34 | 3.51 (1.28) | 3.49 (1.58) |  |  |  |
| ABBV-552 1 mg | 22 | 3.80 (1.33) | 3.98 (2.14) | 0.25 (0.36) | [-0.46, 0.97] | 0.488 |
| ABBV-552 5 mg | 20 | 3.53 (1.09) | 3.23 (1.18) | -0.20 (0.37) | [-0.93, 0.54] | 0.601 |
| ABBV-552 15 mg | 31 | 3.95 (1.55) | 3.76 (1.59) | -0.03 (0.33) | [-0.68, 0.63] | 0.936 |
| **Female** | 134 |  |  |  |  |  |
| Placebo | 27 | 3.52 (1.20) | 3.63 (1.62) |  |  |  |
| ABBV-552 1 mg | 38 | 3.33 (1.12) | 3.16 (1.49) | -0.33 (0.27) | [-0.85, 0.20] | 0.219 |
| ABBV-552 5 mg | 41 | 3.62 (1.42) | 3.22 (1.69) | -0.60 (0.26) | [-1.12, -0.08] | **0.025** |
| ABBV-552 15 mg | 28 | 3.50 (1.38) | 3.54 (1.92) | -0.13 (0.28) | [-0.70, 0.43] | 0.636 |
| **Age < 65 years** | 33 |  |  |  |  |  |
| Placebo | 12 | 3.08 (0.90) | 3.13 (1.80) |  |  |  |
| ABBV-552 1 mg | 5 | 2.30 (0.45) | 1.90 (0.65) | -0.30 (0.41) | [-1.15, 0.55] | 0.474 |
| ABBV-552 5 mg | 7 | 3.07 (0.67) | 2.36 (1.14) | -0.87 (0.36) | [-1.61, -0.12] | **0.024** |
| ABBV-552 15 mg | 9 | 3.72 (1.75) | 3.61 (1.73) | -0.40 (0.35) | [-1.11, 0.31] | 0.261 |
| **Age 65 to ≤ 75 years** | 100 |  |  |  |  |  |
| Placebo | 19 | 3.39 (1.34) | 3.39 (1.69) |  |  |  |
| ABBV-552 1 mg | 29 | 3.40 (0.97) | 3.41 (1.55) | 0.01 (0.34) | [-0.66, 0.68] | 0.986 |
| ABBV-552 5 mg | 24 | 3.71 (1.21) | 2.83 (1.01) | -0.81 (0.35) | [-1.51, -0.10] | **0.025** |
| ABBV-552 15 mg | 28 | 3.63 (1.46) | 3.54 (1.48) | -0.04 (0.34) | [-0.72, 0.63] | 0.900 |
| **Age ≥ 75 years** | 108 |  |  |  |  |  |
| Placebo | 30 | 3.77 (1.25) | 3.82 (1.42) |  |  |  |
| ABBV-552 1 mg | 26 | 3.85 (1.40) | 3.81 (2.03) | -0.12 (0.34) | [-0.80, 0.56] | 0.726 |
| ABBV-552 5 mg | 30 | 3.62 (1.50) | 3.73 (1.79) | 0.07 (0.33) | [-0.58, 0.72] | 0.834 |
| ABBV-552 15 mg | 22 | 3.89 (1.44) | 3.82 (2.09) | -0.08 (0.36) | [-0.79, 0.63] | 0.826 |
| **Race: White** | 189 |  |  |  |  |  |
| Placebo | 47 | 3.65 (1.26) | 3.72 (1.59) |  |  |  |
| ABBV-552 1 mg | 52 | 3.60 (1.15) | 3.43 (1.55) | -0.26 (0.22) | [-0.69, 0.18] | 0.245 |
| ABBV-552 5 mg | 48 | 3.53 (1.29) | 3.07 (1.46) | -0.56 (0.22) | [-1.01, -0.12] | **0.013** |
| ABBV-552 15 mg | 42 | 3.57 (1.34) | 3.37 (1.34) | -0.28 (0.23) | [-0.73, 0.18] | 0.236 |
| **Race: Not White** | 51 |  |  |  |  |  |
| Placebo | 14 | 3.07 (1.05) | 2.96 (1.45) |  |  |  |
| ABBV-552 1 mg | 8 | 2.88 (1.51) | 3.63 (3.03) | 0.92 (0.65) | [-0.39, 2.23] | 0.164 |
| ABBV-552 5 mg | 13 | 3.81 (1.44) | 3.77 (1.74) | 0.01 (0.56) | [-1.12, 1.15] | 0.980 |
| ABBV-552 15 mg | 16 | 4.25 (1.76) | 4.47 (2.41) | 0.24 (0.56) | [-0.90, 1.37] | 0.674 |
| *Abbreviations:* ANCOVA, analysis of covariance; CI, confidence interval; SD, standard deviation; SE, standard error. | | | | | | |
| *Note:*ANCOVA includes treatment group and stratification factor as main effects and the baseline as a covariate; interaction and main effect *P* values (nominal) are based on type II sum of squares. | | | | | | |
| ^a^Includes all randomized participants who received at least 1 capsule of study drug and had at least 1 post-baseline ADAS-Cog 14 score. | | | | | | |
|  | | | | | | |

| **Supplementary Table 3. Exposure-Response Simulated Change From Baseline** | | |
| --- | --- | --- |
|  | **ADAS-Cog 14 Week 12** | **CDR-SB Week 12** |
|  | **Simulated Change from Baseline** | **Simulated Change from Baseline** |
| **Treatment** | **Median (95% Prediction Interval)** | **Median (95% Prediction Interval)** |
| Placebo | -1.70 (-2.70, -0.79) | 0.04 (-0.32, 0.41) |
| 1 mg QD | -1.51 (-2.36, -0.71) | -0.15 (-0.39, 0.15) |
| 5 mg QD | -1.49 (-2.13, -0.69) | -0.14 (-0.40, 0.16) |
| 15 mg QD | -1.07 (-2.18, 0.04) | -0.15 (-0.40, 0.09) |
| *Abbreviations:* ADAS-Cog 14, 14-item Alzheimer's Disease Assessment Scale-Cognitive Subscale; CDR-SB, Clinical Dementia Rating Scale – sum of boxes; QD, once daily. | | |
